# IRcall and IRclassifier: two methods for flexible detection of intron retention events from RNA-Seq data

**DOI:** 10.1186/1471-2164-16-S2-S9

**Published:** 2015-01-21

**Authors:** Yang Bai, Shufan Ji, Yadong Wang

**Affiliations:** 1 School of Computer Science and Technology, Harbin Institute of Technology, 92 West Dazhi Street, Nan Gang District, 150001 Harbin, China; 2 School of Computer Science and Engineering, Beijing University of Aeronautics and Astronautics, 43 Xueyuan Road, HaiDian District, 100083 Beijing, China

## Abstract

**Background:**

The emergence of next-generation RNA sequencing (RNA-Seq) provides tremendous opportunities for researchers to analyze alternative splicing on a genome-wide scale. However, accurate detection of intron retention (IR) events from RNA-Seq data has remained an unresolved challenge in next-generation sequencing (NGS) studies.

**Results:**

We propose two new methods: IRcall and IRclassifier to detect IR events from RNA-Seq data. Our methods combine together gene expression information, read coverage within an intron, and read counts (within introns, within flanking exons, supporting splice junctions, and overlapping with 5' splice site/ 3' splice site), employing ranking strategy and classifiers to detect IR events. We applied our approaches to one published RNA-Seq data on contrasting *skip *mutant and wild-type in *Arabidopsis thaliana*. Compared with three state-of-the-art methods, IRcall and IRclassifier could effectively filter out false positives, and predict more accurate IR events.

**Availability:**

The data and codes of IRcall and IRclassifier are available at http://mlg.hit.edu.cn/ybai/IR/IRcallAndIRclass.html

## Background

Alternative splicing of precursor messenger RNA (pre-mRNA) produces different mRNA isoforms from a single genic locus during gene expression, resulting in functional complexity and diversity of proteins for higher eukaryotic organisms [1-3]. Alternative splicing has four main patterns that include cassette exon skipping (ES), alternative 5' and 3' splice site (ASS), mutually exclusive exon splicing (MXE) and intron retention (IR) [4]. Among those four alternative splicing patterns, genome-wide intron retention (IR) detection is a popular research topic in biology.

Traditional methods which analyze microarray data, have provided a rich source of information for IR event detection [5]. However, the hybridization-based technology employed by microarray is largely restricted to existing genome sequence knowledge, with a limited range of quantification [6]. With the development of next-generation sequencing technology, RNA-Seq, which extends the analysis of previous unidentified genes and splicing variants, is rapidly outperforming microarrays for genome-wide studies [6,7]. With various statistical and computational strategies, many recent studies have analyzed RNA-Seq data for IR event detection, including ExpressionPlot [8], MATS [9], Wang's Framework [10], IRFinder [11] and etc. ExpressionPlot [8] cuts off low-density introns and adopts Fisher's exact test or Chi-squared test to quantify IR events, using only read counts (within introns/ flanking exons). Another framework MATS [9] calculates the Bayesian posterior probabilities of IR to assess their difference between treatment and control conditions, using only read counts (supporting splice junctions/ within introns). Besides, an analysis pipeline based on de novo mapping using BLAT [12] is introduced by [10], which detects the complete retention of an intron in a transcript. However, BLAT employs some low quality reads for read alignment, which would bring in noises [13]. Recently, IRFinder [11] uses read counts within introns to estimate the difference of read coverage between treatment and control conditions by adopting Audic and Claverie Test [14]. However, as for read coverage calculation, besides uniquely mapped reads, IRFinder employs the reads mapped to multiple positions in the genome, which would bring in noises.

**Figure 1:**
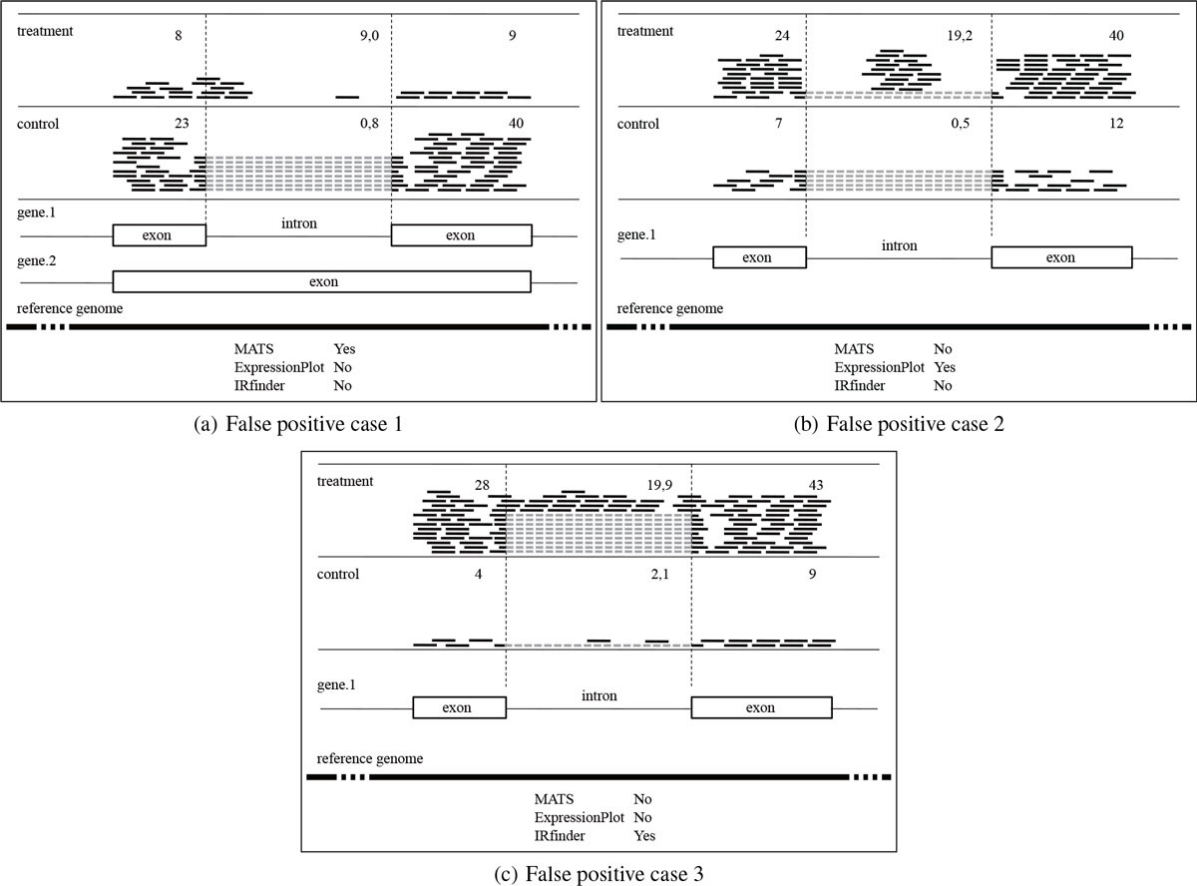
**False positive IR events (Short black bars stand for reads with numbers indicating read counts)**. (a) More reads are overlapping with 5' or 3' splice site, but less reads are locating within an intron; (b) Many reads are clustering within a specific intronic inside region; (c) The gene expression between treatment and control conditions is different.

Existing methods for IR event detection usually omit some significant features (i.e. gene expression information). They depend on only read counts (within an intron, within flanking exons, and supporting splice junction) to estimate IR events between treatment and control conditions [8-11]. They omit the gene expression information and read coverage within an intron or use some un-uniquely mapped reads and low quality reads, which would involve false positive data, reducing prediction precision. As illustrated in Figure 1, there exist three common false positive cases: (1) More reads are overlapping with 5' or 3' splice site, but less reads are locating within an intron; (2) Many reads are clustering within a specific intronic inside region; (3) The gene expression between treatment and control conditions is different, where the read counts (within introns, within flanking exons, supporting splice junctions) should have been similar if there is no difference on gene expression. As for these three false positive cases, MATS and ExpressionPlot falsely predict case (1) and (2) respectively, as they do not consider read distribution and coverage within introns; while IRFinder falsely predicts case (3) as it does not consider gene expression difference. Therefore, we are motivated to take in more features to design more precise prediction methods for IR event detection.

In this paper, we propose two efficient and effective methods: Intron Retention call (IRcall) and Intron Retention classifier (IRclassifer) for of IR event detection from RNA-Seq data. Compared with existing IR event detection methods, IRcall and IRclassifier have four major contributions:

First, besides traditional features for IR event detection, IRcall and IRclassifier employ new features, including read counts overlapping with 5' splice site/ 3' splice site, and gene expression between treatment and control conditions, which help to filter out false positives caused by different gene expression; Second, IRcall employs ranking strategy, with a new formula to calculate IR scores, which avoids bias and declines false positives; Third, IRclassifier employs machine learning techniques, based on J48 Decision Tree and Random Forests, for IR event detection. IRclassifier selects training examples by incorporating the predictions from three state-of-the-art approaches, and constructs 17 features between treatment and control conditions to represent introns in higher-dimensional spaces; Last but not least, experimenting the published RNA-Seq data of *skip *mutant and wild-type in *Arabidopsis thaliana *[10], IRcall and IRclassifier could identify both known and novel IR events, with high prediction precisions. Moreover, IRclassifier based on J48 Decision Tree and Random Forests could predict IR events with a precision of 98.5% and 99.0%, respectively.

## Methods

To overcome limitations of existing approaches, our idea is to utilize more features, employing ranking strategy and machine learning methods for IR event detection.

### Input data

The inputs of IRcall and IRclassifier include the alignment results of RNA-Seq data and the reference annotation. The raw RNA-Seq reads between treatment and control conditions are aligned to the reference genome sequences by TopHat2 [15], and the resulting alignments are stored and sorted in BAM files, defined by [16]. The reference annotation provides the locations of introns, exons and genes. As for the raw RNA-Seq read alignment, the parameters of TopHat2 are set as follows: *min − anchor − length *= 4, *min − intron − length *= 40, *max − intron − length *= 2000, *min − segment − intron *= 40, *max − segment − intron *= 2000, *min − coverage − intron *= 40, and *max − coverage − intron *= 2000.

### Extract features

**Figure 2:**
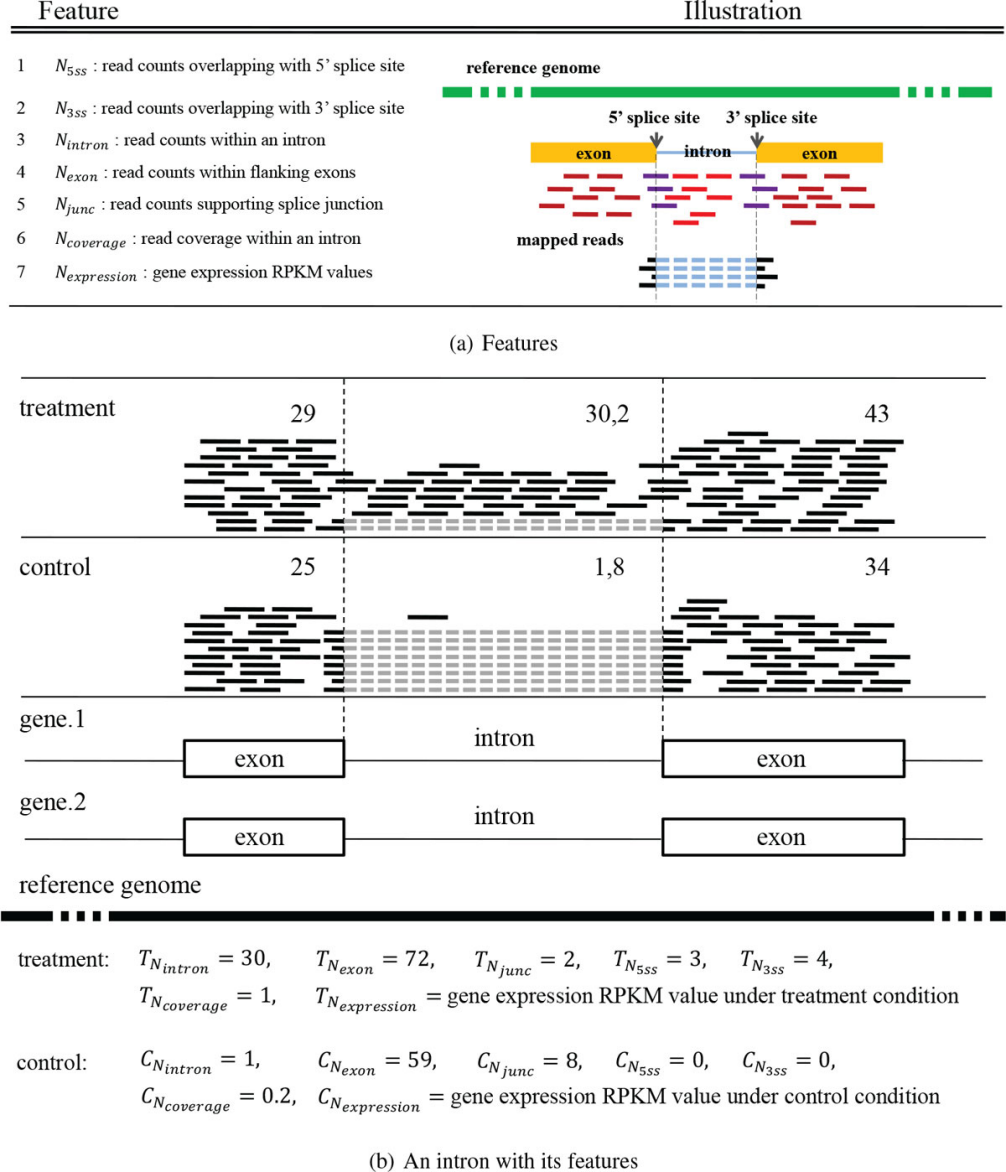
**Features and their illustration**. (a) 7 Features. Red(Dark red/ Purple) short bars stand for the unique mapped reads within the intron (within flanking exons, overlapping with 5'/ 3' splice site); while black short bars connected by blue dash lines stand for the splitted reads supporting splice junctions. (b) An intron with its features between treatment and control conditions.

In each sample of RNA-seq data, we extract 7 features (Figure 2(a)), including read counts within an intron (*N_intron_*), read counts within flanking exons (*N_exon_*), read counts supporting splice junctions (*N_junc_*), read counts overlapping with 5' splice site (*N*_5*ss*_), read counts overlapping with 3' splice site (*N*_3*ss*_), read coverage within an intron (*N_coverage_*) and gene expression information (*N_expression_*).

For *N_intron_*, *N_exon_*, *N_junc_*, *N*_5*ss *_and *N*_3*ss*_, we count the original reads within an intron, within flanking exons, supporting splice junctions, overlapping with 5' splice site and 3' splice site, respectively. As for *N_coverage_*, we calculate the percentage of covered positions (with one read at least) within an intron. In addition, we employ Cuffdiff [17] to calculate gene expression RPKM values (RPKM: Reads per kilo base per million), denoted by *N_expression_*.

As shown in Figure 2(b), we will get 7 features $T_{N_{{}_{intron}}}$, $T_{N_{exon}}$, $T_{N_{Junc}}$, $T_{N_{5ss}}$, $T_{N_{3ss}}$, $T_{N_{coverage}}$ and $T_{N_{expression}}$ for treatment condition, and 7 features $C_{N_{intron}}$, $C_{N_{{}_{exon}}}$, $C_{N_{Junc}}$, $C_{N_{5ss}}$, $C_{N_{3ss}}$, $C_{N_{coverage}}$ and $C_{N_{{}_{expression}}}$ for control condition. Those 14 features form the feature space of an intron for IR event detection.

### IRcall

**Figure 3:**
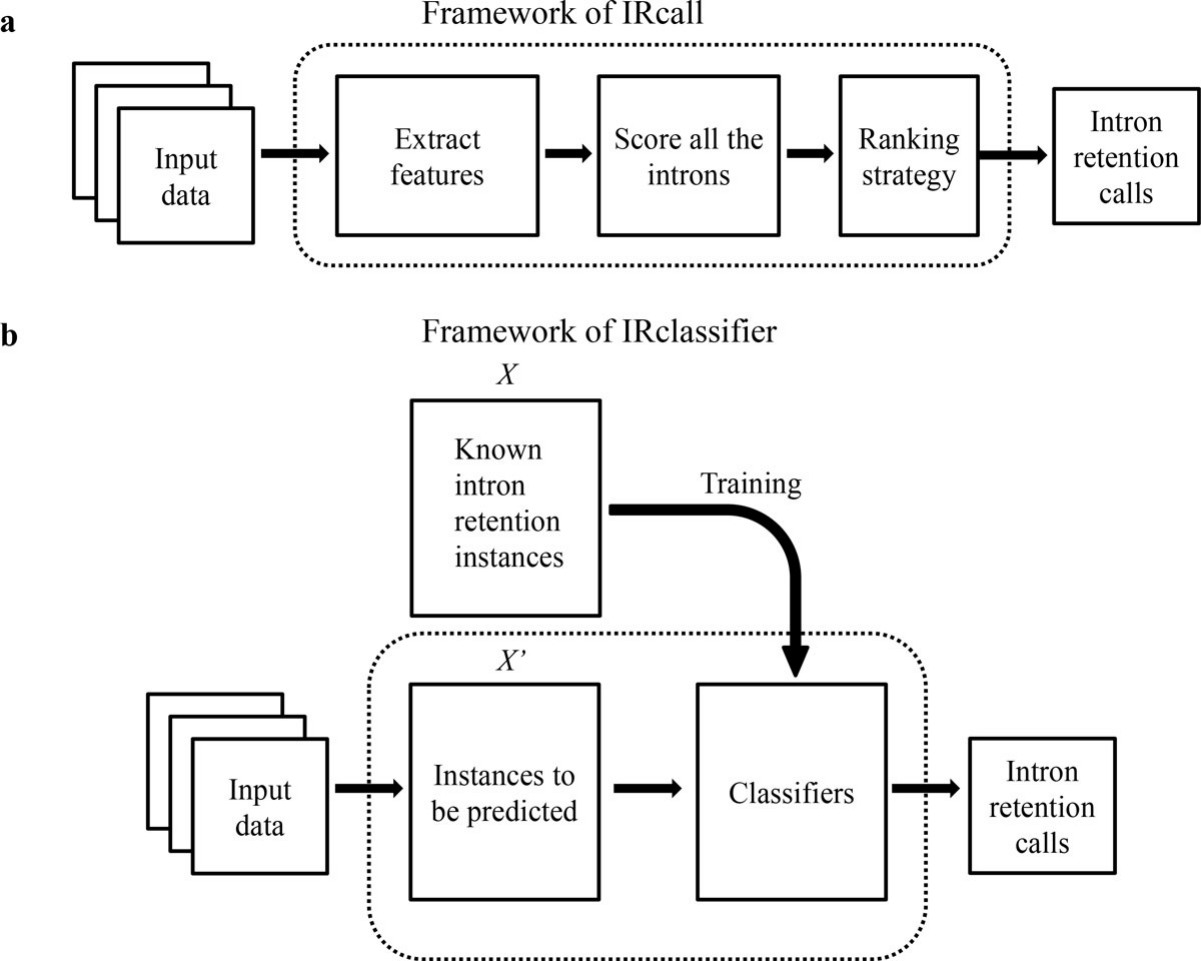
**Frameworks of IRcall and IRclassifier**.

The framework of IRcall is illustrated in Figure 3(a): the input data (bam files, and the reference annotation) are firstly analyzed to extract 14 features, representing each intron between treatment and control conditions; then low quality introns are removed according to the criteria in Table 1; after that, the IR score for each intron is calculated according to the features; finally, the introns are ranked by IR scores in descending order, with top-*n*% introns returned as IR events. Note that *n*% is the user-specified threshold; and an intron is retained for further process if it satisfies all criteria in Table 1. The criteria come from some domain knowledge and experiments [8,11].

**Table 1:** Intron Removal Criteria.

$T_{N_{5ss}}$	>	3
$T_{N_{3ss}}$	>	3
$T_{N_{coverage}}$	>	0:9
$T_{N_{exon}}$	>	1
$T_{N_{expression}}$	>	10
$T_{N_{{}_{intron}}}$	>	1
$C_{N_{{}_{expression}}}$	>	10
$C_{N_{Junc}}$	>	1
$C_{N_{{}_{exon}}}$	>	1
$\frac{T_{N_{expression}}}{C_{N_{{}_{expression}}}}$	<	$log_{2}\left(\frac{3}{2}\right)\;\;\text{or}\;\;\;\frac{T_{N_{expression}}}{C_{N_{{}_{expression}}}}>log_{2}\left(\frac{2}{3}\right)\;\;$

We propose a novel IR score formula to reflect the IR changes between treatment and control conditions, on three aspects: (a) ratio of read counts within an intron to flanking exons (NDIE); (b) ratio of read counts within an intron to read counts supporting splice junctions (NDIJ); (c) read coverage within an intron (NDIC). The IR score takes the weighted average of the three aspects, formulated as:

(1) $$\begin{array}{c} IRScore=\omega_{1}*NDIE+\omega_{2}*NDIJ+\omega_{3}*NDIC\\ \;\;\left(\omega_{1}+\omega_{2}+\omega_{3}=1\right) \end{array}$$

In our experiments, we set equal weights to *NDIE*, *NDIJ *and *NDIC*, thus $\omega_{1}=\omega_{2}=\omega_{3}=\frac{1}{3}$. The *NDIE*, *NDIJ *and *NDIC *are calculated as follows:

First, in all treatment and control samples, *IE*, *IJ *, and *IC *of each intron are calculated to reflect the ratio of read counts within an intron to flanking exons, the ratio of read counts within an intron to read counts supporting splice junctions, and read coverage within an intron, by formula (2) (3) (4):

(2) $$IE=\left(\frac{N_{intron}}{N_{{}_{exon}}}\right)$$

(3) $$IJ=\left(\frac{N_{intron}}{N_{junc}}\right)$$

(4) $$IC=e^{N_{coverage}}$$

Second, *DIE*, *DIJ *and *DIC *are calculated to reflect the changes in *IE*, *IJ *, and *IC *between treatment and control conditions (logarithm is taken to handle zero values in denominators), by formula (5) (6) (7). Note that *DIE*, *DIJ *and *DIC *should be larger than zero; otherwise, the intron instance is filtered out as noise.

(5) $$DIE=log_{2}\left(\frac{IE_{{}_{treatment}}}{IE_{{}_{control}}}\right)$$

(6) $$DIJ=log_{2}\left(\frac{IJ_{{}_{treatment}}}{IJ_{{}_{control}}}\right)$$

(7) $$DIC=log_{2}\left(\frac{IC_{{}_{treatment}}}{IC_{{}_{control}}}\right)$$

Finally, as the value scales of *DIE*, *DIJ *and *DIC *are different, we normalized them to the same scale of 0 to 1, by dividing their corresponding maximum value *MAXDIE*, *MAXDIJ *and *MAXDIC*, resulting in *NDIE*, *NDIJ *and *NDIC*, as shown in formula (8) (9) (10):

(8) $$NDIE=\frac{DIE}{MAXDIE}$$

(9) $$NDIJ=\frac{DIJ}{MAXDIJ}$$

(10) $$NDIC=\frac{DIC}{MAXDIC}$$

As IRcall defines proper intron removal criteria (in Table 1) and IR score formula, it well considers read counts (within introns, within flanking exons, supporting splice junctions, and overlapping with 5' splice site/ 3' splice site), read coverage within an intron, and gene expression information, between treatment and control conditions. Hence, IRcall could effectively filter out false positives and improve prediction precision.

### IRclassifier

IRclassifier is a framework drawing on machine learning ideas to facilitate better identification of IR events from RNA-Seq data, which contains three main steps (Figure 3(b)): first, the training set is constructed by incorporating the predictions of ExpressionPlot [8], MATS [9] and IRFinder [11], with 17 significant features to interpret each instance. The instances are saved in feature matrix ***X ***, labeled with "yes" and "no", indicating positive and negative ones, respectively; second, the J48 Decision Tree (J48) classifier and Random Forests (RF) classifier are trained respectively, with 10-fold cross-validation to avoid over-fitting; finally, new RNA-Seq data are sent to the two classifiers respectively for IR event prediction.

Our training data set are constructed based on the results of IR event identification, by MATS, ExpressionPlot and IRFinder. The instances hit by at least two of the three predicting algorithms are taken as "positive" ones, labeled by "yes", while those hit by none of the algorithms are taken as "negative" ones, labeled by "no". Thus, 741 positive instances and 3525 random negative instances are selected as the training set. The instance is interpreted by 17 features: 14 features from treatment and control samples (Figure 2) and 3 features *DIE*, *DIJ *, and *DIC *(defined in section Methods).

As for classifiers, we employ J48 Decision Tree and Random Forests algorithms from Weka [18] to conduct two independent predictions. J48 classifier forms rules from pruned partial decision trees built by C4.5 heuristics [19], which minimizes the number of tree levels and tree nodes to maximize data generalization. During tree building, information gain is adopted for feature selection, such that feature of largest information gain is chosen for the next splitting. RF classifier [20] constructs independent decision tree classifiers by bagging and random feature selection, and aggregates the outputs of each decision tree in RF to produce one final prediction by a majority vote of the trees. Here, the RF classifier is constructed with 10 decision trees, each selecting 5 random features. To avoid overfitting, both J48 classifier and RF classifier employ 10-fold cross-validation.

## Results and discussion

We use real RNA-seq data http://www.ncbi.nlm.nih.gov/sra?term=SRP008262 to detect IR events, which are comprise of *skip *mutant and wild-type in *Arabidopsis thaliana *[10], including two replicated *skip *mutant samples (skip1 and skip2) and two replicated wild-type samples (wt1 and wt2). As for the reference, we take *Arabidopsis thaliana *TAIR9 genome, with sequences and annotations ftp://ftp.arabidopsis.org/home/tair/Genes/TAIR9_genome release/. The performance of IRcall and IRclassifier are studied against state-of-the-art IR prediction methods ExpressionPlot [8], MATS [9] and IRFinder [11].

### Data normalization and experimental setup

As the numbers of mapped reads in the two *skip *mutant samples (skip1 and skip2) and two wild-type samples (wt1 and wt2) are different (28465249 reads in skip1, 20849546 reads in skip2, 44865242 reads in wt1, and 18343654 reads in wt2), we take the minimum read number 18343654 as the standard normalization scale (SNS). Then all read counts that construct features are divided by its sample's overall read number and multiplied by SNS for normalization. The sum of normalized read counts in skip1 and skip2 (wt1 and wt2) is taken as the read count for treatment (control) sample. Then all relevant features and scores are calculated according to the algorithms in Section Methods.

**Figure 4:**
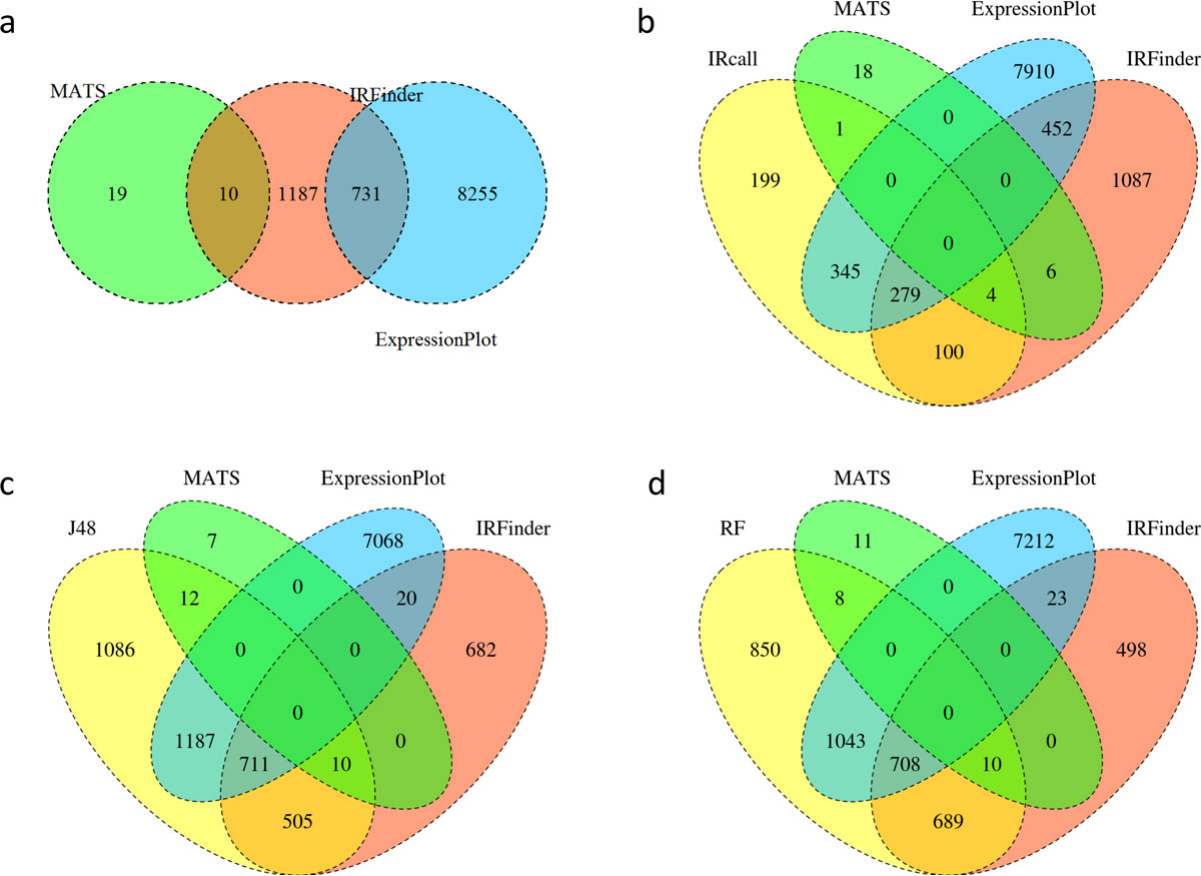
**IR Event Predictions**. (a) Predictions of MATS, ExpressionPlot and IRFinder; (b) Predictions of IRcall, MATS, ExpressionPlot and IRFinder; (c) Predictions of IRclassifier (J48 Decision Tree), MATS, ExpressionPlot and IRFinder; (d) Predictions of IRclassifier (RF), MATS, ExpressionPlot and IRFinder.

As for evaluation reference, we get all positive IR event predictions (see Figure 4(a)) of ExpressionPlot [8], MATS [9], and IRFinder [11], with higher statistical significance (***P ***-value *<*0.05). In total, there are 10202 positive IR event predictions, with 29 from MATS, 8986 from ExpressionPlot, and 1928 from IRFinder.

### IRcall performance

In the first group of experiments, IRcall is taken to detect differential IR events from RNA-Seq data on *skip *mutant and wild-type. For all of the 158580 introns in *Arabidopsis thaliana *TAIR9 reference annotation, we firstly filtered out the introns not satisfying the intron removal criteria of IRcall (see Table 1), resulting in 928 IR event candidates. Then the IR scores of those 928 IR event candidates are calculated, according to which the candidates are sorted in descending order.

To evaluate the performance of IRcall, we compared the 928 IR event candidates of IRcall with the positive predictions of MATS, ExpressionPlot and IRFinder. As illustrated in Figure 4(b), the 928 IR event candidates of IRcall have 199 new predictions, and 5, 624, 383 overlaps with MATS, ExpressionPlot, and IRFinder, respectively. From the new predictions and each overlap, we randomly picked one case to display by IGV software [21] and [22], to study whether the introns are retained. As shown in Figure 6, our new prediction intron 1 of AT4G26080.1 (d) is retained between treatment and control conditions, in the similar way as those in overlaps with MATS (a), ExpressionPlot (b) and IRFinder (c). That is, our IRcall could predict known and novel IR event candidates.

**Figure 5:**
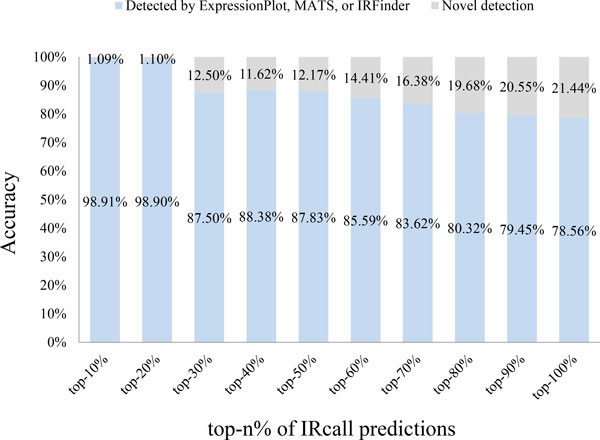
**Accuracy of the top-*n*% IRcall predictions**.

However, those overlaps in Figure 4(b) only demonstrate the worst case of IRcall, as the IR score and ranking strategy have not been applied. To verify that the top-ranked IR event candidates tend to be true IR events, we studies the accuracy of IRcall top-*n*% predictions with the evaluation reference, that is, the 10202 positive predictions of MATS, ExpressionPlot and IRFinder. As shown in Figure 5, with the increase of returned IR events number (from top-10% to top-100%), the prediction accuracy decreases. That is to say, the top-ranked IRcall predictions tend to have higher accuracy (i.e. 98.91% for top-10% predictions), thus the IR score and ranking strategy proposed by IRcall are effective for IR event detection. Therefore, biologists are suggested taking top-ranked IR events for wet-lab verification.

**Figure 6:**
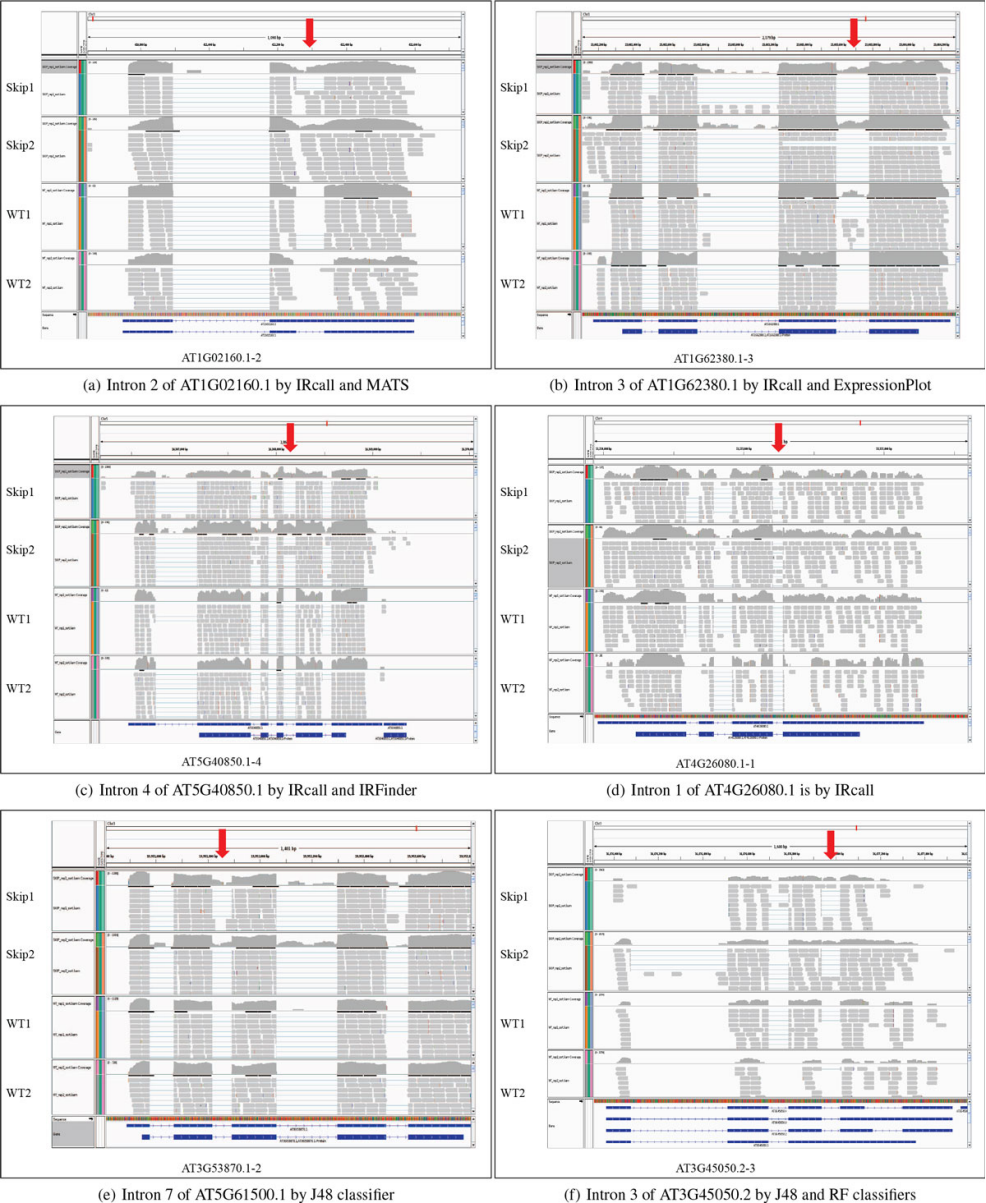
**Pre-mRNAs with predicted IR events (red arrows indicating retained introns)**.

### IRclassifier performance

As for IRclassifier, the instances hit by at least two of the three algorithms MATS, ExpressionPlot and IRFinder are taken as our positive training instances, labeled by "yes". As shown in Figure 4(a), the positive predictions of IRFinder and MATS have 10 overlaps, IRFinder and ExpressionPlot have 731 overlaps, while MATS and ExpressionPlot have none overlap. Thus, we get 741 instances for our positive training set. As for the negative training set, we randomly take 3525 introns hit by none of the above three algorithms, labeled by "no". 10-fold cross-validation is employed to avoid over-fitting and evaluate prediction accuracy for both J48 and RF classifiers. As shown in Table 2, the J48 classifier is of 99.1% true negative rate (specificity), 96.0% true positive rate (sensitivity) and 98.5% accuracy; while the RF classifier is of 99.7% true negative rate, 96.3% true positive rate and 99.0% accuracy.

**Table 2:** IRclassifier Performance.

Classifier	Acc	Sp	Sn	AUC
J48	98.5%	99.1%	96.0%	97.2%
RF	99.0%	99.7%	96.3%	99.8%

Acc: Accuracy, Sp: Specificity, Sn: Sensitivity, AUC: Area under ROC curve.

In addition, the predictions of IRclassifier are evaluated with the reference predictions of MATS, ExpressionPlot and IRFinder. As shown in Figure 4(C) and 4(D), the J48 classifier predicts 1086 new IR events, and 22 MATS, 1898 ExpressionPlot, 1226 IRFinder overlaps; while the RF classifier predicted 850 new IR events, and 18 MATS, 1751 ExpressionPlot, 1407 IRFinder overlaps. Among the new IRclassifier predictions, we randomly choose two cases displayed by IGV software in Figure 6: (e) intron 7 of AT5G61500.1 (predicted by J48 classifier) in *skip *mutant was retained compared with wild-type; and (f) AT3G45050 (predicted by J48 and RF classifiers) was different spliced between treatment and control conditions, where transcript 1 of AT3G45050 (AT3G45050.1) was expressed under treatment condition, but transcript 2, 3, and 4 of AT3G45050 (AT3G45050.2, AT3G45050.3 and AT3G45050.4) are expressed under control condition.

### Feature ranking

To examine how each individual feature affects IR event prediction, we adopt the information gain for feature ranking. As each feature comes from both treatment and control samples, we take the value of *treatment/control *for information gain calculation in that IR events usually exist where the difference between treatment and control samples is significant. As shown in Table 3, features with higher information gains are relatively more relevant to IR event detection. The results show that the read counts within an intron, read counts overlapping with 5' and 3' splicing site are the most influential features for IR event detection.

**Table 3:** Feature ranking.

Features(treatment/control)	Information gain
read counts within an intron	0.4038
read counts overlapping with 3 splicing site	0.3948
read counts overlapping with 5 splicing site	0.3917
read coverage within an intron	0.2496
read counts supporting splice junction	0.2343
read counts within flanking exons	0.0713
gene expression RPKM values	0.0527

## Conclusions

In this paper, we overcome the limitations of traditional statistical and computational methods for IR event identification from RNA-Seq data, by proposing two novel algorithms, IRcall and IRclassifier, to detect the IR events between treatment and control conditions. In our methods, new features are employed to filter out false positives effectively. IRcall takes 14 features and defines a novel formula *IRscore *for candidate IR event ranking. From experiments, 98.91% of IRcall's top-10% high ranked IR event candidates have been verified by other methods. As a machine learning method for IR event detection, IRclassifier employs 17 features, and gets 98.5% and 99.0% recognition precisions, with J48 Decision Tree classifier and Random Forests classifier, respectively. Therefore, we believe that our IRcall and IRclassifier are effective to detect IR events between treatment and control conditions from RNA-Seq data, delivering valuable information and tools for alternative splicing research.

In future, instead of using TopHat2 for junction alignment, we will consider assessing the influence of different junction alignment methods on the performance of IRcall and IRclassifier. Besides, we plan to use IRcall and IRclassifier to detect IR events from paired-end sequencing data.

## Competing interests

The authors declare that they have no competing interests.

## Authors' contributions

YB, SJ and YW conceived and designed the algorithm and experiments. YB implemented the algorithm and finished the experiments. All authors rote and revised the manuscript.

## Declaration

Publication of this article was funded by the Natural Science Foundation of China [Nos: 61173085 and 61102149] and the National High-Tech Research and Development Program (863) of China [Nos: 2012AA020404, 2012AA02A602 and 2012AA02A604].

This article has been published as part of *BMC Genomics *Volume 16 Supplement 2, 2015: Selected articles from the Thirteenth Asia Pacific Bioinformatics Conference (APBC 2015): Genomics. The full contents of the supplement are available online at http://www.biomedcentral.com/bmcgenomics/supplements/16/S2
